# Neural Correlates of Gender Face Perception in Transgender People

**DOI:** 10.3390/jcm9061731

**Published:** 2020-06-03

**Authors:** Alessandra Daphne Fisher, Jiska Ristori, Giovanni Castellini, Carlotta Cocchetti, Emanuele Cassioli, Stefano Orsolini, Carolina Sensi, Alessia Romani, Francesca Mazzoli, Agnese Cipriani, Valdo Ricca, Linda Vignozzi, Maria Pia Viggiano, Mario Mascalchi, Mario Maggi, Gioele Gavazzi

**Affiliations:** 1 Andrology, Women’s Endocrinology and Gender Incongruence Unit, Careggi University Hospital, 50139 Florence, Italy; carlotta.cocchetti@gmail.com (C.C.); alessiaromani@hotmail.it (A.R.); francesca.mazzoli@stud.unifi.it (F.M.); agne.cipr@gmail.com (A.C.); mario.mascalchi@unifi.it (M.M.); mario.maggi@unifi.it (M.M.); 2 Mario Serio” Department of Experimental and Clinical Biomedical Sciences, University of Florence, 50121 Florence, Italy; jiska.ristori@unifi.it (J.R.); linda.vignozzi@unifi.it (L.V.); 3 Department of Neuroscience, Psychology, Drug Research, Child Health, University of Florence, 50135 Florence, Italy.; giovanni.castellini@unifi.it (G.C.); emanuele.cassioli@gmail.com (E.C.); carolina.sensi@gmail.com (C.S.); valdo.ricca@unifi.it (V.R.); mariapia.viggiano@unifi.it (M.P.V.); 4 Department of Electrical, Electronic, and Information Engineering “Guglielmo Marconi”, University of Bologna, 47521 Cesena, Italy; stefano.orsolini@gmail.com; 5 IRCCS SDN, 80142 Naples, Italy; gioelegavazzi@gmail.com

**Keywords:** gender face perception, gender incongruence, gender dysphoria, transmen, transwomen

## Abstract

To date, MRI studies focused on brain sexual dimorphism have not explored the presence of specific neural patterns in gender dysphoria (GD) using gender discrimination tasks. Considering the central role of body image in GD, the present study aims to evaluate brain activation patterns with 3T-scanner functional MRI (fMRI) during gender face discrimination task in a sample of 20 hormone-naïve transgender and 20 cisgender individuals. Additionally, participants were asked to complete psychometric measures. The between-group analysis of average blood oxygenation level dependent (BOLD) activations of female vs. male face contrast showed a significant positive cluster in the bilateral precuneus in transmen when compared to the ciswomen. In addition, the transwomen group compared to the cismen showed higher activations also in the precuneus, as well as in the posterior cingulate gyrus, the angular gyrus and the lateral occipital cortices. Moreover, the activation of precuneus, angular gyrus, lateral occipital cortices and posterior cingulate gyrus was significantly associated with higher levels of body uneasiness. These results show for the first time the existence of a possible specific GD-neural pattern. However, it remains unclear if the differences in brain phenotype of transgender people may be the result of a sex-atypical neural development or of a lifelong experience of gender non-conformity.

## 1. Introduction

Gender incongruence (GI) is defined by a marked and persistent discrepancy between an individual’s experienced gender and the assigned sex [1]. When this condition is associated with a clinically significant distress or impairment in social, occupational or other important areas of functioning, it is referred to as gender dysphoria (GD) [2]. Individuals whose gender identity does not completely and/or permanently match their sex characteristics may describe themselves as trans or transgender. On the other hand, individuals whose gender identity does match their sex characteristics are referred to as cisgender.

The potential impact of biologic and social/cultural factors in the etiology of GI/GD remains under debate. However, biology seems to play a major role [3,4]. In the last years, the idea of the sexual dimorphic brain as the anatomic substrate of psychosexual development has been more widely pursued and research has focused on the influence and shaping role of genes and gonadal hormones on sexual differentiation of the brain [5]. In particular, gonadal steroids may influence the development of brain structures and circuits through an organizational effect during early development and an activational effect later in life [3,6].

Brain structural and functional differences—resulting from the interaction of genes and sex hormones with the developing brain—are thought to be the developmental substratum of gender identity and factors interfering with this complex process may be involved in or at least favor, GI/GD [4].

Morphologic and functional brain characteristics of transgender individuals have been investigated in vivo with magnetic resonance imaging (MRI), in order to substantiate this etiological hypothesis with a biologic background. Regarding brain morphology, several studies supported this hypothesis especially in transwomen, showing a pattern in line with gender identity with regards to cortical thickness, regional gray matter volumes and white matter microstructure [7,8,9,10,11]. On the other hand, findings seem to be more conflicting in transmen when evaluating morphologic brain characteristics. Particularly, some authors reported a gray matter pattern superposable to the assigned sex [7,12], while other studies found structural characteristics similar to those observed in cisgender males [13,14]. Additionally, in transmen, morphologic patterns different from both cisgender groups have been described in literature [8,15,16,17]. The discrepant results of the aforementioned studies could be partly attributed to several limitations including inhomogeneity of selected samples concerning sexual orientation, GD levels, age and previous hormonal treatments [18].

Given the fact that cognitive abilities are often gender-specific, some studies evaluated differences in functional brain characteristics during task-based MRI studies. During a verbal fluency task, Soleman et al. [19] did not find significant differences in brain activation pattern between transgender adolescents and cisgender controls. Contrastingly, regarding visuospatial cognitive functioning, two studies reported atypical brain activation during a mental rotation task in transgender individuals, different from that observed in the cisgender control group [18,20]. In addition. differences in resting-state brain networks in transgender women compared to cisgender ones have been reported [21,22]. Moreover, combining both resting-state functional connectivity and behavioral data, gender identity in transgender and in cisgender persons has been recently examined, showing that machine learning algorithms could predict distinct brain connectivity patterns in cis and trans people [23].

Further studies have focused on functional MRI connectivity evaluating differences between transgender and cisgender persons in cerebral networks involved in own body perception, given its importance in GD [14,15,24,25,26,27]. Several authors hypothesized that GD could be based on the disconnection of fronto-parietal networks involved in the processing of own body image. This may lead transgender individuals to be unable to incorporate typical body characteristics of their gender assigned at birth into their own body representation in the brain [28]. Particularly, a central role seems to be played by the default mode network, which represents a connectivity network involved in mind-wandering and self-referential thinking [29] and by the salience network, which could be involved in processing different stimuli from own body [30,31]. Some evidences suggest that differences in networks connectivity related to body perception and self-identification may represent a neurobiologic correlate of GD.

To the best of our knowledge, no MRI study has explored the presence of possible specific neural activation pattern in transgender people by using a gender discrimination task. For this reason, considering the central role of body image in GD development, in the present study we evaluated brain activation patterns related to face gender discrimination in a sample of hormone-naïve transgender and cisgender individuals by using fMRI.

## 2. Methods

### 2.1. Participants

Forty subjects, namely 20 cisgender controls (10 cismen, 10 ciswomen) and 20 transgender individuals (10 transmen, 10 transwomen) of similar age (mean ± SD age = 28.45 ± 5.17 and 29.55 ± 10.90 years, respectively for cisgender and for transgender individuals; *t* = 0.408 *p* = 0.68), took part in the experiment.

The 20 trans individuals belonged to a consecutive series of subjects referring for the first time to the center for GI/GD at the University of Florence. They were enrolled in the present study if the following inclusion criteria were met:

- Age older than 18 years;
- Diagnosis of GD made by mental care providers experienced in GD during consultations and by assessing DSM 5 criteria [2,32].

The exclusion criteria included:

- The use at any point in life of hormonal therapy.
- Gender affirming surgery performed.
- Illiteracy.
- Mental retardation.
- Disorder of sexual development (DSD).
- Severe and unstable psychiatric conditions (e.g., psychotic disorders, depressive disorder with suicidal ideation) assessed by mental health professionals experienced in GD during consultations and by assessing DSM 5 criteria [2]

The cisgender group was enrolled by means of local advertisement at the University of Florence, when the following inclusion criteria were met: age older than 18 years, absence of GD or psychiatric disorders. The exclusion criteria included:

- The use in the previous six months of any hormonal treatment and of any psychiatric medication;
- Illiteracy;
- Mental retardation;
- DSD;
- Severe and unstable psychiatric conditions (e.g., psychotic disorders, depressive disorder with suicidal ideation);
- Pregnancy or current lactation.

All participants had normal or corrected-to-normal visual acuity and normal hearing, by self-report. Participants were screened to ensure that they satisfied MRI safety requirements and showed no structural brain abnormalities on T1 or FLAIR MRI sequences (see below) obtained before the fMRI task.

### 2.2. Sociodemographic and Psychometric Evaluations

All participants underwent a physical examination, with measurement of height, weight and body mass index (BMI).

In addition. they were asked to complete the body uneasiness test (BUT) [33], the gender identity/gender dysphoria questionnaire for adolescents and adults [34] and the symptom checklist-90 revised (SCL-90-R) [35].

The BUT is a self-rating scale exploring different areas of body-related psychopathology, including dissatisfaction with the body and its weight (weight phobia), avoidance, compulsive control behavior (compulsive self-monitoring), experience of separation and strangeness from the body (depersonalization) and specific worries about certain body parts, characteristics or functions. The subjects were asked to rate 34 different body image experiences (BUT A) and 37 body parts (BUT B) on a six-point Likert scale (from 1 = never to 6 = always), indicating how often they happen to dislike each experience or each body part. Higher scores indicate greater body uneasiness. BUT scores were analyzed by considering the total score of the test (global severity index), the number of disliked body parts (positive symptoms total, PST) and the mean intensity of dislike of all disliked body parts (positive symptom distress index, PSDI) [33].

The gender identity/gender dysphoria questionnaire for adolescents and adults (GIDYQ-AA) is a 27-item questionnaire evaluating GD (17). Each item is rated on a 5-point response scale, considering the past 12 months as time frame. The response options are: always, often, sometimes, rarely or never, coded 1 to 5, respectively. Lower scores are associated with higher GD, being a score of three suggested as critical threshold for GD diagnosis. Internal coherence was satisfactory for the Italian validated version (α value of about 0.97) [34].

Furthermore, levels of psychopathologic distress were specifically investigated by means of the Italian version of the symptom checklist (SCL-90-R) [35], which was answered for the week preceding the clinical assessment. The 90 items of the questionnaire are rated on a five-point Likert scale (from 0 to 4) and are grouped together into nine domains (somatization, obsessive–compulsive thoughts, interpersonal sensitivity, depression, anxiety, hostility, phobic anxiety, paranoid conceptions and psychotic behavior). In this study, we utilized the general severity index (SCL-GSI), indicating the overall psychological distress.

To evaluate sexual orientation dimensionally, a visual analog scale (VAS) was used, rating 0 when sexual attraction was exclusively towards people of the same perceived gender (i.e., towards men in cis- and transmen and towards women for cis- and transwomen) to 10, when sexual attraction was exclusively towards the opposite gender from the perceived one (i.e., towards women for cis- and transmen and towards men for cis- and transwomen) [36].

Written informed consent was obtained from each participant and was approved by the local ethical committee (2013/0016117) in agreement with the 1964 Helsinki declaration and its later amendments or comparable ethical standards.

### 2.3. Rationale and Description of Experimental Paradigm

The intense body-related distress experienced by some transgender people [37,38,39] may result in focusing on gender-specific body characteristics. Body sexual dimorphic parts may represent indeed for some transgender people the inner source of their sufferance and frequently of their low social acceptance. Taking this in mind, we speculated that the gender discrimination task, which requires focalizing on gender dimorphic facial features, may activate a more intense emotional reaction related to the painful remind of one’s gender incongruence.

**Figure 1:**
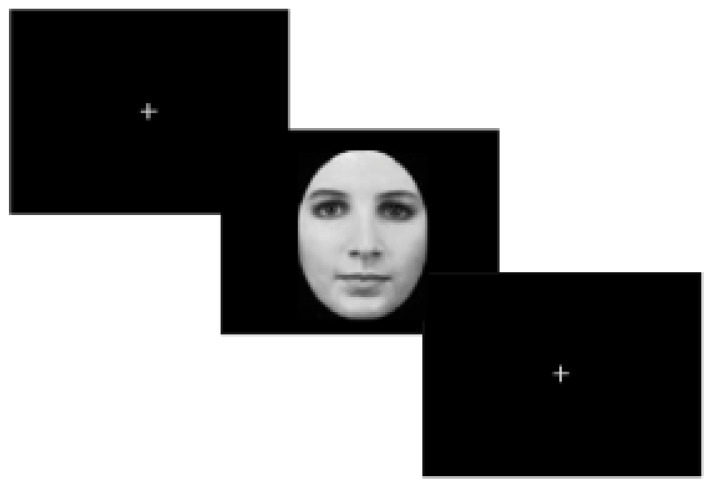
Task. Each trial started with a cross of fixation positioned in the center of the screen and was displayed for 3 s. Then, a face stimulus (50% male; 50% female) replaced it and was presented for 3 s. Participants had to observe the face displayed in the monitor and report its gender. At the end of each trial there was a fixation cross located in the center of the screen for 6 s (rest phase).

Stimuli were administered by means of psycho-toolbox 3 (20) and custom Matlab code. A desktop computer was employed to display the visual stimuli. Participants viewed stimuli on a MRI-compatible display system (SensaVue fMRI, Invivo Corporation, Gainesville, FL, USA) with a mirror attached to the head coil. The experiment was composed by two sessions: a training session and a task session. The training session consisted in a block of five trials, to ensure understanding of the instructions. The task session was composed by 40 trials. The protocol was a Gender Face Detection task (hereafter GFD). Each GFD trial started with a cross of fixation positioned in the center of the screen for 3 s and was followed by a face stimulus (50% of times male; 50% of times female) for 3 s (Figure 1).

Participants had to observe the face displayed in the monitor and discriminate its gender by pressing one of two possible buttons associated to the face gender perceived (male or female). At the end of each trial there was a rest phase of six seconds where a fixation cross was located in the center of the screen. To avoid any possible effect of learning or cognitive strategies, stimuli were equally in number and randomly presented. The models of faces employed in this experiment were selected from a validated database (Karolinska database) [40]. The faces were transformed in black and white, equated in luminance and the hair contour was removed with Photoshop. Two male and two female original faces with neutral expression were chosen. We created for each face the respective face of the opposite gender by means of FaceApp to obtain 4 male and 4 female faces matched for identity for a total of 8 different stimuli. FaceApp is a software developed by a Russian company, Wireless Lab, which uses GANs networks to generate highly realistic transformations of faces’ pictures. The distinctive features which make the identity unique remain unchanged, whereas the algorithm allows to transform a face to make it smile, look younger, older or change gender [41,42,43].

### 2.4. MRI Data Acquisitions.

MRI acquisitions were performed on a 3T scanner (Ingenia, Philips Healthcare, Best, The Netherlands) equipped with Omega HP gradients with maximum amplitude of 45 mT/m and slew rate of 200 T/m/s for each axis. All subjects underwent 3D T1-weighted imaging and fMRI, using a 32-phased-array-element head coil.

T1-weighted MR images were acquired with a sagittal high-resolution 3D sequence (repetition time [TR] = 8 ms, echo time [TE] = 3.7 ms, inversion time [TI] = 925.6 ms, flip angle [FA] = 8°, slice thickness = 1 mm, field of view [FOV] = 240 mm × 240 mm, number of slices = 191, matrix size = 352 × 352).

T2-weighted 3-dimensional fluid-attenuated inversion recovery (FLAIR)–volume isotropic turbo spin–echo acquisition images were acquired on the coronal plane (repetition time [TR] = 8000 ms, echo time [TE] = 355 ms, inversion time [TI] = 2400 ms, variable [10°–180°] flip angle, echo–train length = 110, slice thickness = 1 mm, field of view = 256 × 256 mm, matrix = 232 × 232, 155–175 sections, number of excitations = 1, sensitivity encoding factor = 3.0, fat suppression with spectral selection attenuated inversion recovery; acquisition time 5 min and 28 s).

For the fMRI experiment we employed a T2*-weighted echo-planar imaging (EPI) sequence (TR/TE = 3000/35 ms, FA = 90°, slice thickness = 3.5 mm, FOV = 240 mm × 240 mm, number of slices = 42, matrix size = 240 × 240). One hundred and sixty five scans were acquired, for a total acquisition time of about 8 min, from which the first 5 scans were discarded.

### 2.5. Data Analysis

Volumes acquired with fMRI were analyzed using the FMRIB Software Library (www.fmrib.ox.ac.uk/fsl). Canonical preprocessing was applied (first 5 time points removed, slice-time correction with custom timings, motion correction and intensity normalization). As filtering steps, we adopted the following: temporal high-pass with cutoff at 50 s; spatial smoothing using a 4 mm full width half-maximum Gaussian kernel. Co-registration of fMRI images to the individual high-resolution T1-weighted image was performed using a 6-degree of freedom registration. The individual high resolution T1-weighted images were co-registered to the standard space Montreal Neurological Institute 152 (MNI152) brain with an affine transformation (12 degree of freedom) followed by a nonlinear transformation. fMRI images were co-registered to the MNI152 standard space using the transformation previously computed when co-registering the individual high-resolution T1-weighted images to the MNI152 standard space.

Time points in the fMRI data set that were affected by large motion, namely displacement >1.5 mm of the absolute mean displacement, were identified from motion correction parameters (motion correction FMRIB’s linear image registration tool) and accounted for in a confound matrix at the subject-level analysis. Each stimulus delta functions sequence was convolved with a double gamma hemodynamic response function, whereas the temporal derivative was included in the model and temporal filtering applied.

To explore activity related to the gender face perception, a general linear model contrast was set at subject-level analysis to analyze volumes correspondent to the time interval of face presentation where the two explanatory variables (EV) were set as volumes of female presented face and male presented face.

Each model EV was convolved with a double gamma hemodynamic response function, whereas temporal derivatives were included, and temporal filtering applied. This contrast was assessed for within and between groups’ analyses.

To establish between-group differences of the five comparisons of major interest (cismen vs. ciswoman, transmen vs. cismen, transwomen vs. ciswomen, cismen vs. transwomen and ciswomen vs. transmen), we used an unpaired *t*-test with a mixed effects model taking into account Bonferroni correction of p-value for the considered multiple comparisons.

Because the experimental design involved randomized intervals stimuli, we reduced autocorrelation in the data applying voxel-wise pre-whitening. To establish between-group differences, we used an unpaired t-test with a mixed effects model. All group analyses were performed in the MNI152 standard space T1-weighted template. For all statistical analyses, the resulting Z (Gaussianized T/F) statistic images were thresholded using clusters determined by Z >2.3 and a (corrected) cluster significance threshold of *p* < 0.01. To anatomically map the significant clusters in the resulting Z statistic images with labels of maximum probability, we used the Harvard–Oxford cortical and subcortical structural probabilistic atlases [44,45]. Moreover, to localize the occipital face area and Face Fusiform Area we used coordinates defined in previous studies [46,47].

Continuous variables were reported as mean ± standard deviation. The independent sample *t*-test was used to compare continuous variables. Univariate analysis of variance (ANOVA) was used to compare the continuous variables among groups, entering age and BMI as a covariate, when appropriate.

Post hoc paired contrasts with Tukey’s B tests were performed for the pairwise comparison among the groups. Pearson’s correlation was used to evaluate the associations between different variables within each group. Bonferroni correction was applied for multiple comparisons. Differences between groups were evaluated in multivariate models (adjusting for BMI and age) by means of analysis of covariance (ANCOVA). Finally, linear and logistic regression analyses were used for multivariate analysis (adjusting for BMI and age) whenever appropriate. All analyses were performed using SPSS version 25 (SPSS, Inc., Chicago, IL, USA).

## 3. Results

### 3.1. Sociodemographic and Clinical Characteristics

Table 1 reports the socio-demographic and clinical variables of groups and their differences in an age-adjusted model. transwomen showed significantly lower BMI than other groups (*p* = 0.035). No differences were found among groups in terms of sexual orientation (*p* = 0.155). In addition. no differences between transmen and transwomen were found in terms of GD intensity and onset, according the GIDYQ-AA and the clinical interview (all *p* > 0.05).

### 3.2. fMRI

**Figure 2:**
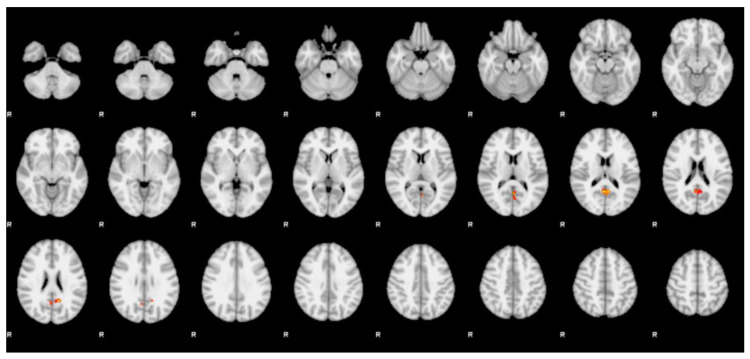
Ciswomen vs. transmen. Between-group analysis during the face presentation (female → male) shows clusters of significantly (*p* < 0.01) higher blood oxygen level dependent (BOLD) effect in transmen group when compared with ciswomen. The activations are included in the precuneus. Cluster formation threshold was set at 2.3 of Z statistic value. Coordinates are reported in Montreal Neurological Institute space.

The within-group analysis of differential BOLD activations of female vs. male face contrast did not reveal any significant cluster of activation. However, the between-group analysis of differential BOLD activations of the female vs. male face contrast showed several statistically significant clusters. One cluster of positive statistic sign (Z > 2.3, *p* < 0.01) was observed in the bilateral of the precuneus in the transmen group when compared with the ciswomen group (Figure 2 and Table 2).

**Figure 3:**
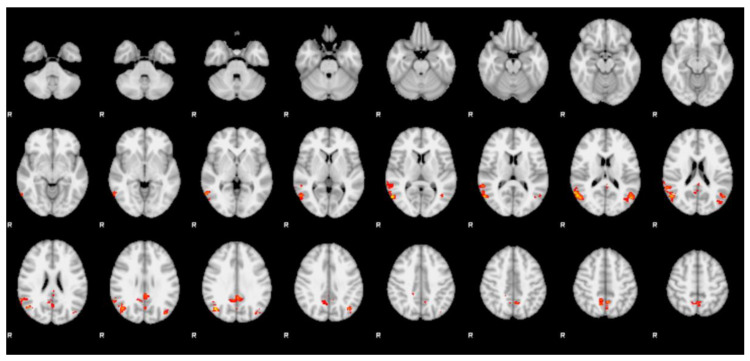
Cismen vs. transwomen. Between-group analysis during the face presentation (female → male) shows clusters of significantly (*p* < 0.01) higher blood oxygen level dependent (BOLD) effect in transwomen group when compared with cismen. They include bilaterally the precuneus, the posterior cingulate gyrus, the angular gyrus and the lateral occipital cortices (including the occipital face area). Coordinates are reported in Montreal Neurological Institute space.

Four clusters of positive statistic sign (Z > 2.3, *p* < 0.01) were observed in the transwomen group when compared with the cismen group (Figure 3 and Table 3).

The first cluster was located in the right lateral occipital cortex (including the occipital face area—OFA) and the right angular gyrus (r-AG). The second cluster comprised the bilateral posterior division of the cingulate gyrus (PCG) and the precuneus. The third cluster included the left lateral occipital cortex (including the OFA) and the left angular gyrus (l-AG). The fourth cluster was located bilaterally in the precuneus and extended in the left lateral occipital cortex.

All other between group analyses did not demonstrate any significant cluster of differential activation. No significant results were reported for the negative sign statistics.

### 3.3. Psychometric Evaluations

In the entire sample, several measures of general psychopathology and body uneasiness, as well as anthropometric measures were considered. Differences among groups were adjusted for age and BMI, which may affect results (Table 4).

**Figure 4:**
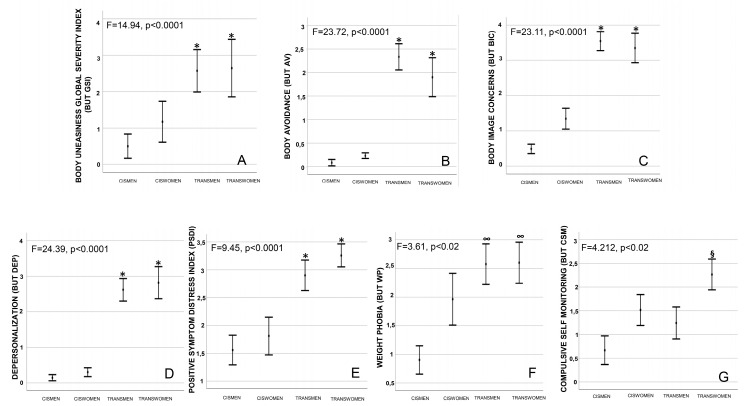
**(A)** Body uneasiness global severity index (BUT GSI), (**B**) body avoidance (BUT AV), (**C**) body image concerns (BUT BIC), (**D**) depersonalization (DEP), (**E**) positive symptom distress index (PSDI), (**F**) weight phobia (BUT WP) and (**G**) compulsive self-monitoring (BUT CSM) according to different groups as derived by ANCOVA and Post hoc Tukey’s B test after adjustment for age and body mass index. * transwomen and transmen vs. ciswomen and cismen; ∞ transwomen and transmen vs. cismen; § transwomen vs. other groups. ANCOVA= Analysis of covariance.

When BUT was analyzed, trans people showed significantly higher body uneasiness levels compared with the cisgender groups (*p* < 0.0001; Figure 4A). Accordingly, scores of several BUT subscales (including “avoidance”, “body image concerns”, “depersonalization” and “positive symptoms distress index”) were significantly higher in trans groups compared to the cis ones (all *p* < 0.02, Figure 4B–E for BUT AV, BIC, DEP and PSDI, respectively). In addition. groups showed significant differences in terms of weight phobia subscales (BUT WP), with transwomen and transmen reporting higher scores compared to cismen (*p* < 0.02, Figure 4F). Finally, transwomen showed significant higher compulsive self-monitoring scores (BUT CSM) compared to cismen (*p* < 0.02, Figure 4G).

Considering body uneasiness related to different body parts (BUT B, Table 5), transwomen showed significantly higher distress towards sexual dimorphic characteristics of the face (i.e., forehead, brows, nose, mouth, chin, moustache, beard), whereas transmen towards breast, when compared to all other groups (all *p* < 0.005). Other correlations are showed in Table 5.

### 3.4. Association between fMRI Imaging Results and Body Uneasiness Levels

Because body uneasiness is age-and BMI-correlated [33], all the following results were also adjusted for the aforementioned variables.

**Figure 5:**
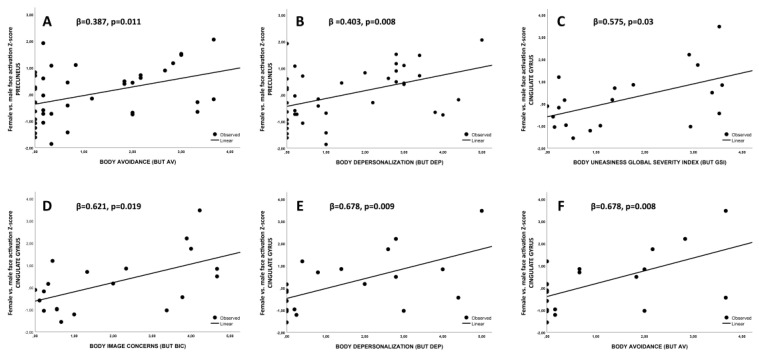
Association between precuneus and posterior cingulate gyrus ROI Z-scores with body uneasiness test-related scales (**A**,**B**) and (**C**)–(**F**), respectively). ROI= region of interest.

Since a higher differential activation in the precuneus was observed in both transmen and transwomen (vs. ciswomen and cismen, respectively), the whole sample was considered for the associations between precuneus ROI Z-score and BUT. Indeed, a positive association between precuneus ROI Z-score and body avoidance (BUT AV) as well as depersonalization (BUT DEP) scores (both *p* < 0.02; Figure 5A,B, respectively) was observed.

As a higher differential activation in the posterior cingulate gyrus, in the angular gyrus and in the lateral occipital cortices was observed only in transwomen group when compared to cismen one, only these groups were considered for the following associations. In particular, the posterior cingulate gyrus Z-score was associated with body uneasiness global score (BUT GSI), body image concerns (BIC), depersonalization (BUT DEP) and avoidance (BUT AV) subscales was observed (all *p* < 0.02, Figure 5C–F, respectively). Beta values are reported in Figure 5. Associations between BUT GSI and BIC were not confirmed after corrections for multiple comparisons even though they showed a high significant level (*p* = 0.017 and 0.009, respectively).

**Figure 6:**
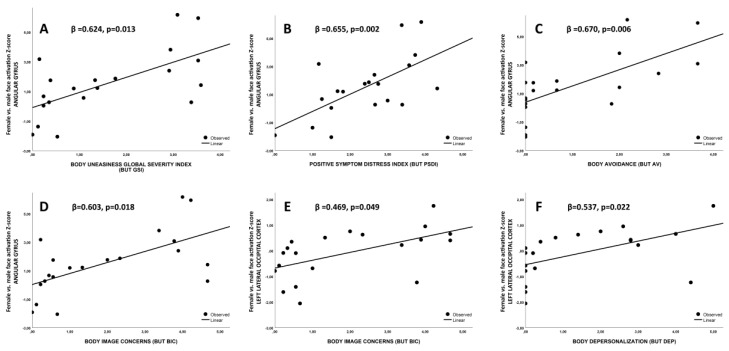
(**A**)–(**D**) Association between angular gyrus ROI Z-scores with body uneasiness test related scales; (**E**,**F**) association between left lateral occipital cortex Z-score with body uneasiness test scales. ROI= region of interest.

In addition, angular gyrus ROI Z-score was associated with body uneasiness global score (BUT GSI), as well with body uneasiness towards body parts total score (BUT PSDI) (both *p* < 0.02, Figure 6A,B, respectively). angular gyrus ROI Z-scores was associated with BUT subscales related to body image avoidance behavior (AV) and body image concerns (BIC; both *p* < 0.02; Figure 6C,D, respectively). Moreover, considering individual body parts, angular gyrus ROI-Z score was associated with sexual dimorphic facial characteristics, including brows, eyes, nose, chin, moustache and beard (all *p* < 0.02). When left lateral occipital cortex Z-score was considered, a positive association with both BUT BIC and depersonalization scales (BUT DEP) were found (both *p* < 0.05, Figure 6E,F, respectively). Beta values are reported in Figure 6.

**Figure 7:**
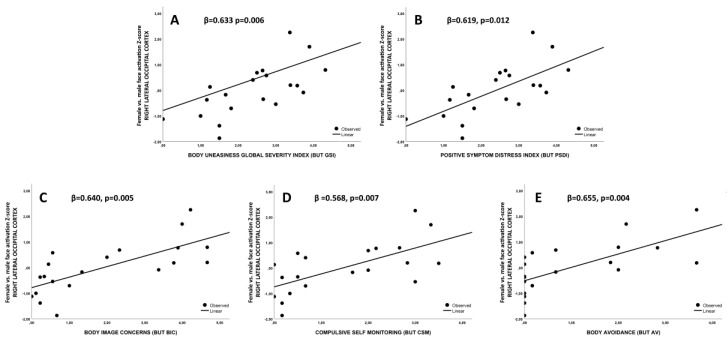
(**A**)–(**E**) Association between right lateral occipital cortex Z-score and body uneasiness test related scales.

Finally, right lateral occipital cortex Z-Score was associated with BUT global score (BUT GSI, 0.633 *p* = 0.006, Figure 7A), with body uneasiness towards body parts total score (BUT PSDI, 0.619, *p* = 0.012, Figure 7B) as well as with BUT BIC, CSM and AV subscales (all *p* < 0.02; Figure 7C–E, respectively). Beta values are reported in Figure 7.

## 4. Discussion

This is the first neuroimaging study evaluating the face gender discrimination and its psychopathologic correlates in a sample of transgender people, compared to a sample of cisgender ones. By using validated questionnaires and fMRI, we demonstrated for the first time specific neural activation patterns in transgender people. However, it remains unclear if differences in the brain phenotype of transgender people are the result of a sex-atypical neural development or of a lifelong experience of gender non-conformity. The strengths of the present study include (i) a comprehensive design, integrating neural correlates with psychological functioning; (ii) the homogeneity of the transgender people sample with respect to age, onset of feelings of GD, sexual orientation and hormonal treatment (all hormone-naïve) [5]. The main limitation is in the cross-sectional design of the study.

The main results are the following: (i) the transmen group showed a higher differential activation in the precuneus, as compared with the ciswomen one; (ii) the transwomen group, when compared to the cismen one, showed a higher differential activation in the precuneus, in the posterior cingulate gyrus, in the angular gyrus and in the lateral occipital cortices (at variance from the transmen vs. the ciswomen groups); (iii) according to psychometric evaluation (BUT), transwomen showed significantly higher distress towards sexual dimorphic characteristics of the face than transmen; (iv) differential activation in the precuneus, angular gyrus, lateral occipital cortices and posterior cingulate gyrus areas was associated with higher levels of body uneasiness.

### 4.1. Association between Precuneus Activation and Psychometric Evaluations

Among the aforementioned results, the most relevant is the higher differential activation in the precuneus for transgender groups when compared to the cisgender ones of the same assigned sex at birth (transwomen vs. cismen; transmen vs. ciswomen). precuneus is a superior parietal region recruited by a wide spectrum of tasks like visuo-spatial abilities, episodic memory retrieval and self-processing operations [48]. However, more recently, it has been proposed that the precuneus may also be implicated in the face perception elaboration. Indeed, the precuneus seems to be activated by identity recognition of familiar and famous persons as well as during the encoding of one’s own identity [49]. In addition. the precuneus seems to be involved in empathic judgements, as reported by the Ochsner’s fMRI study [50]. Along this line, the significant higher precuneus differential activation in the transgender groups on the face discrimination task, suggests that this region may play a crucial role in the gender identification process in transgender people, but not in the cisgender ones. We can postulate that this higher differential activation may be related to a more intense emotional involvement in transgender people while discriminating faces’ gender. Indeed, body sexual dimorphic characteristics represent for some transgender people the inner source of their sufferance and a painful remind of their gender incongruence. The experienced body-related distress may also result in a selective attention, with an intense focus on gender-specific body characteristics. In line, the gender discrimination task requires focalizing on gender dimorphic facial features. This may lead to a negative emotional arousal in transgender people due to an associative mechanism between gender-dimorphic features of the displayed faces and their own.

Indeed, trans people show significant higher levels of body uneasiness (BUT) compared to cisgender ones (see Figure 4). This result confirms previous studies showing transgender individuals being more dissatisfied with their body than cisgender persons [37,51], suggesting that the body is the primary source of their suffering. The possible more intense emotional involvement in transgender vs. cisgender people during a gender identification task seems here to be corroborated by the positive correlation between the precuneus ROI Z-score with body avoidance (BUT AV) and depersonalization (BUT DEP) BUT subscales (Figure 5). In line, it has been reported that the precuneus is involved in representing different body identities and in discriminating what is perceived as part of the Self from what is perceived as stranger or unfamiliar [52].

An alternative explanation can be hypothesized. Transgender people sufferance may be linked not only to gender incongruence itself, but also to the fear and/or the experience of not being socially accepted. This may be particularly true regarding facial-dimorphism characteristics which are socially visible and on which transgender people may activate a selective focus. Therefore, the hyperactivation of the precuneus may reflect the anticipated and/or experienced discrimination in transgender people in regards of self-recognition and definition.

### 4.2. Associations between Posterior Cingulate Gyrus and Angular Gyrus Activations and Psychometric Evaluations

Notably, the between group analysis of the transwomen group vs. the cismen group (differently from the transmen vs. the ciswomen groups) revealed additional clusters of statistically significant higher activations beyond the precuneus, including posterior cingulate gyrus, the angular gyrus and the lateral occipital cortices. The posterior cingulate gyrus activation in a face perception task is not surprising. This region resides below the precuneus and has often been reported to be co-activated with the precuneus in face perception tasks assessing discrimination between famous and less famous faces [49].

The angular gyrus is located in the posterior part of the inferior parietal lobule and is activated in face-voice integration during person recognition [53,54]. In particular, Lee et al. [55] demonstrated that face components can be reconstructed from angular gyrus fMRI activity patterns, suggesting a striking role of this region in the memory and perceptual identification of faces. Moreover, fMRI studies reported that angular gyrus is involved in perception and processing of the ‘dispositions and intentions of other individuals’, i.e., social evaluation of faces [56,57] and is a core region in conceptual processing [58].

The significant positive association of both posterior cingulate gyrus and angular gyrus Z-score with body uneasiness levels here observed (Figure 5C–E and Figure 6A–D) further supports the hypothesis of a more intense emotional involvement during gender recognition task in trans people compare to cis ones. In addition. the previously reported involvement of angular gyrus in social evaluation of faces [58] may acquire a more intense significance in trans individuals (especially transwomen), who often focus on low social (and/or interiorized) acceptance of their facial sexually dimorphic features [59]. Indeed, angular gyrus ROI-Z score showed an association with sexually dimorphic facial characteristics, including brows, eyes, nose, chin, moustache and beard.

### 4.3. Associations between Lateral Occipital Cortices Activations and Psychometric Evaluations

Considering the activations of the lateral occipital cortices, it is worth to note that the clusters identified by our analysis included the occipital face area (OFA), but not the Fusiform Face Area (FFA). OFA and FFA have crucial functions in face perception. Lesion studies have shown that both the OFA and the FFA are necessary to assign an identity to an observed face. In fact, a malfunctioning of one of the two can lead to apperceptive prosopagnosia [60,61,62]. In particular, FFA is involved in the elaboration of face parts and their spatial configurations [63], whereas OFA is selectively employed to analyze the single face parts, e.g., mouth, nose or eyes shapes [64,65]. The higher differential activation of OFA in transwomen when compared to cismen (differently from the transmen vs. the ciswomen groups) suggests that during the cognitive process of gender identification of an observed face, these subjects recruit a lateral occipital network based on the elaboration of face elements shape. In line with this possibility, body uneasiness towards facial parts (BUT B) showed significant higher scores in transwomen with respect to all other groups. In particular, transwomen reported significantly higher distress towards sexual dimorphic characteristics of the face (i.e., forehead, brows, nose, mouth, chin, moustache, beard) when compared to transmen (Table 4). Accordingly, the intensity of the activation of these brain regions (both left and right lateral occipital cortices) was significantly associated with higher levels of dissatisfaction with physical appearance (Figure 6E,F and Figure 7A–D).

Notably the combination between body uneasiness (BUT) and the coupling of angular gyrus and OFA activation in transwomen, with respect to cismen, strongly support the possibility that the analysis of the face parts is at the basis of the gender judgement in this category of transgender subjects. As a possible explanation for both the association between higher emotional attitude towards face observation in transwomen and its association with specific cerebral activation can be related with more intense shaping effect of male sex steroids during puberty on facial features. Indeed, during pubertal development, testosterone accentuates typical facial dimorphic characteristics (i.e., large jaws, prominent mandible and zygoma) as well as Adam’s apple, increasing the bodily incongruence with the perceived gender in socially exposed physical areas. Pressure to conform to gender stereotypes in western world may be considered an alternative explanation. Therefore, the differences reported in this study could be explained by the effort exerted by transwomen to adhere to the Western female stereotype also by valorizing/hiding facial parts. This may include make-up practices that require a deep inspection of women facial parts. In support, transmen when compared with ciswomen did not show activation in those brain regions related to the inspection of facial parts.

### 4.4. Limitations

Considering the small sample size, the results of the present study should be considered as preliminary. Furthermore, they should be considered in the light of some limitations, such as the use of self-reported measures. The main purpose of this work was to evaluate the neural correlates of face gender discrimination in transgenders. For this reason, we measured the differential activation deriving by the contrast female vs. male faces and not the absolute activations induced by the perception of male or female faces. Since other investigations conducted with different tasks have shown that sexual attraction can affect brain activations [25,66,67], we cannot exclude the contribution of sexual orientation in our results. However, we believe that the sexual orientation may marginally if ever have influenced our results. This convincement is based not only on the contrast we used, but also on the lack of additional areas in the other between groups’ analyses. This notwithstanding, to overcome this limitation, future studies that explores the potential relation between sexual orientation and the face gender discrimination has to be conducted to clarify this issue.

Additionally, in this work we exclusively employed definite male vs female faces to explore the neural correlates of gender face perception. For this reason, we believe that further experiments using more morphing stages will be necessary to elucidate potential differences in face perception of transgender people and allow the investigation of mechanisms underlying this process.

Finally, we admit that based on our results we can only hypothesize that the bilateral precuneus engagement observed in both transgender groups can be crucial in the gender face discrimination. In fact, the precuneus involvement can be associated to cognitive processes that have not been controlled here, like attention, response inhibition or conscious information processing [68,69,70]. Therefore, our experimental design cannot measure how much the precuneus recruitment in the gender face discrimination was due to the higher attention triggered by observing female or male faces, to the presence of inhibitory processes and/or to the conscious information processing related to the face observed.

## 5. Conclusions

Our results show for the first time that GD is associated with specific brain activation patterns during gender face discrimination task. In particular, the precuneus seems to play a crucial role in gender face identity perception in the transgender groups, but not in the cis-gender ones. Additionally, transwomen, with respect to cismen, showed a differential activation of the posterior parietal cortex, the angular gyrus and the lateral occipital cortices (including the OFA, but not the FFA). Notably, these regions are all engaged in the elaboration of face parts and for this reason we can speculate that discriminating faces on the basis of gender may involve a higher attentional demand in transwomen than in cismen. This may be the result of a selective focus on gender-dimorphic facial characteristics by transwomen because of the body uneasiness itself as well as the fear of not being accepted in a transphobic society.

The difference in brain phenotypes of transgender people compared to cisgender controls may suggests a sex-atypical development of the brain. However, further research is needed to clarify if these differences depend on a pre-natal sex-atypical neural development or are the consequence of a lifelong inner and social gender non-conformity experience.

## Figures and Tables

**Table 1:** Summary of means, standard deviations and statistical differences of the main sociodemographic characteristics between cismen, ciswomen, transmen and transwomen as derived by analysis of covariance (ANCOVA) and Post hoc Tukey’s B test after adjustment for age.

	Cismen (*n* = 10)	Ciswomen (*n* = 10)	Transmen (*n* = 10)	Transwomen (*n* = 10)	F	*p*
Height (cm)	179 ± 0.8	163 ± 0.08	161 ± 0.04 **	1.70 ± 0.93	10.91	**<0.0001**
Weight (kg)	**72.30 ± 10.50** ^¥^	56.80 ± 9.04	56.83 ± 5.67	56.70 ± 10.03	9.96	**<0.0001**
Body mass index (kg/m^2^)	22.48 ± 1.98	21.28 ± 2.06	21.78 ± 1.94	**19.65 ± 2.09** ^∞∞^	3.21	**0.035**
Educational level (years of school)	15.40 ± 2.37	16.30 ± 2.63	**11.50 ± 3.84** ^££^	13.70 ± 3.71	4.35	**0.011**
VAS for Sexual orientation	89.26 ± 31.45	94.94 ± 9.75	68.17 ± 41.92	71.41 ± 28.16	1.86	0.155
GIDYQ-AA	4.74 ± 0.11	4.80 ± 0.14	**2.35 ± 0.24** ^∞^	**2.20 ± 0.24** ^∞^	565.58	**<0.0001**
GD onset (years)	–	–	11.40 ± 6.09	9.50 ± 4.58	0.62	0.44

^∞^ transwomen and transmen vs. ciswomen and cismen; ^∞∞^ transwomen vs. cismen; ^¥^ cismen vs. other groups; ** transmen vs. cismen vs. transwomen; ^££^ transmen vs. ciswomen and cismen. VAS = visual analog scale. GIDYQ-AA = gender identity gender dysphoria questionnaire for adolescents and adults; GD = Gender Dysphoria; ANCOVA = Analysis of covariance. Boldfaced numbers highlight statically significant differences between groups.

**Table 2:** Clusters of significantly (*p* < 0.01) higher differential activation in the transmen Group when compared with ciswomen for female→male face contrast.

Cluster	Z-stat	x (mm)	y (mm)	z (mm)	Region (Harvard)
1	3.01	−14	−54	26	left precuneus cortex
	3.01	0	−56	14	left precuneus cortex
	3	−6	−56	16	left precuneus cortex
	2.89	−2	−60	16	left precuneus cortex
	2.82	−2	−62	8	left precuneus cortex
	2.74	4	−56	22	right precuneus cortex

**Table 3:** Clusters of significantly (*p* < 0.01) higher differential activation in the transwomen group when compared with cismen for female → male face contrast.

Cluster	Z-stat	x (mm)	y (mm)	z (mm)	Region (Harvard)
1	3.99	58	−66	8	right lateral occipital cortex
	3.9	44	−68	30	right lateral occipital cortex
	3.71	56	−62	16	right lateral occipital cortex
	3.64	60	−56	16	right angular gyrus
	3.56	48	−64	22	right lateral occipital cortex
	3.52	50	−68	16	right lateral occipital cortex
2	3.44	−2	−46	30	left cingulate gyrus, posterior division
	3.06	−2	−40	28	left cingulate gyrus, posterior division
	2.83	6	−52	32	right cingulate gyrus, posterior division
	2.82	12	−54	34	right precuneus cortex
	2.78	0	−66	26	left precuneus cortex
	2.75	8	−56	24	right precuneus cortex
3	3.58	−52	−66	16	left lateral occipital cortex
	3.14	−40	−68	36	left lateral occipital cortex
	3.06	−48	−58	16	left angular gyrus
	3.02	−38	−76	30	left lateral occipital cortex
	2.84	−42	−64	10	left lateral occipital cortex (l-OFA)
	2.8	−40	−72	18	left lateral occipital cortex (l-OFA)
4	3.38	−2	−56	50	left precuneus cortex
	3.09	10	−58	46	right precuneus cortex
	3.06	10	−50	48	right precuneus cortex
	3.01	12	−58	50	right precuneus cortex
	2.74	−12	−62	60	left lateral occipital cortex
	2.72	−10	−68	58	left lateral occipital cortex

**Table 4:** Summary of means, standard deviations and statistical differences in terms of general psychopathology (SCL-90R), gender dysphoria levels (GIDYQ-AA) and body uneasiness (BUT) between Cismen, Ciswomen, Transmen and Transwomen, as derived by ANCOVA and Post-hoc Tukey B test after adjustment for Age and BMI.

	Cismen (*n* = 10)	Ciswomen (*n* = 10)	Transmen (*n* = 10)	Transwomen (*n* = 10)	F	*p*
**SCL-90 R GSI**	0.30 ± 0.21	0.62 ± 0.42	0.79 ± 0.57	0.97 ± 0.68	2.16	0.16
**GIDYQ-AA GLOBAL SCORE**	4.74 ± 0.11	4.80 ± 0.14	**2.35 ± 0.24** ^∞^	**2.20 ± 0.24** ^∞^	518.82	**<0.0001**
**GIDYQ-AA SUBJECTIVE INDICATOR**	4.86 ± 0.17	4.78 ± 0.17	**2.03 ± 0.17** ^∞^	**2.05 ± 0.32** ^∞^	487.58	**<0.0001**
**GIDYQ-AA SOCIAL INDICATOR**	4.42 ± 0.20	4.77 ± 0.30	**2.92 ± 0.55** ^¥^	**2.46 ± 0.54** ^§^	82.29	**<0.0001**
**GIDYQ-AA SOMATIC INDICATOR**	5.00 ± 0.00	4.97 ± 0.11	**1.40 ± 0.54** ^∞^	**1.50 ± 0.95** ^∞^	141.62	**<0.0001**
**GIDYQ-AA SOCIO-LEGAL INDICATOR**	5.00 ± 0.00	4.95 ± 0.16	**3.20 ± 0.42** ^∞^	**3.05 ± 0.60** ^∞^	70.30	**<0.0001**
**BUT GSI**	0.50 ± 0.47	1.17 ± 0.79	**2.57 ± 0.82** *	**2.65 ± 1.10** *	14.94	**<0.0001**
**BUT WP**	0.90 ± 0.78	1.96 ± 1.43	**2.58 ± 1.12** *	**2.60 ± 1.13** *	3.61	**0.013**
**BUT BIC**	0.49 ± 0.42	1.34 ± 0.93	**3.54 ± 0.85** *	**3.35 ± 1.32** *	23.11	**<0.0001**
**BUT AV**	0.83 ± 0.21	0.23 ± 0.20	**2.33 ± 0.88** *	**1.90 ± 1.31** *	23.72	**<0.0001**
**BUT CSM**	0.67 ± 0.96	1.52 ± 1.03	1.24 ± 1.08	2.27 ± 1.02 ^§^	4.21	**0.012**
**BUT DEP**	0.15 ± 0.27	0.30 ± 0.39	2.62 ± 1.00 *	2.82 ± 1.53 *	24.39	**<0.0001**
**BUT PSDI**	1.56 ± 0.84	1.81 ± 1.08	**2.90 ± 0.87** *	**3.26 ± 0.65** *	9.45	**<0.0001**

^§^ transwomen vs. other groups; ^¥^ cismen vs. other groups; ^∞^ transwomen and transmen vs. ciswomen and cismen; * transwomen and transmen vs. ciswomen and cismen; BMI = Body Mass Index; SCL-90 = symptom checklist-90; GIDYQ-AA = gender identity/gender dysphoria questionnaire for adolescents and adults; BUT = body uneasiness test; GSI = global severity index; WP = weight phobia; BIC = body image concerns; AV = avoidance; CSM = compulsive self-monitoring; DEP = depersonalization; PSDI = positive symptom distress index. Boldfaced numbers highlight statically significant differences between groups.

**Table 5:** Summary of means, standard deviations and statistical differences in dislike of body parts (BUT-B) between male cismen, ciswomen, transmen and transwomen, as derived by ANCOVA and Post hoc Tukey’s B test after adjustment for age and BMI.

BUT Body Parts	Cismen	Ciswomen	Transmen	Transwomen	F	*p*
Height	0.80 ± 1.48	1.20 ± 1.62	2.70 ± 2.16	1.10 ± 1.52	2.76	0.057
Head shape	0.00 ± 0.00	0.50 ± 0.85	0.50 ± 0.71	0.90 ± 1.29	1.89	0.327
Skin	0.80 ± 1.32	1.30 ± 1.42	0.60 ± 0.84	1.60 ± 1.64	1.40	0.257
Hair	0.80 ± 1.57	0.70 ± 1.06	0.20 ± 0.42	1.90 ± 2.18	2.45	0.064
Face shape	0.10 ± 0.32	0.90 ± 1.29	1.30 ± 1.34	1.80 ± 1.40	2.65	0.113
Forehead	0.60 ± 1.26	0.00 ± 0.00	0.20 ± 0.42	**2.20 ± 2.10** ^§^	4.52	**0.009**
Brows	0.30 ± 0.95	0.50 ± 0.71	0.50 ± 0.97	**2.40 ± 1.78** ^§^	5.03	**0.005**
Eyes	0.30 ± 0.95	0.50 ± 0.97	0.90 ± 1.29	1.60 ± 1.65	1.47	0.24
Nose	0.50 ± 0.97	1.30 ± 1.34	1.00 ± 1.15	**3.50 ± 1.58** ^§^	5.97	**0.002**
Lips	0.20 ± 0.63	0.20 ± 0.42	0.40 ± 1.52	1.40 ± 1.45	2.71	0.060
Mouth	0.10 ± 0.32	0.10 ± 0.32	0.40 ± 0.70	**1.40 ± 1.35** ^§^	5.15	**0.005**
Teeth	0.40 ± 0.70	1.30 ± 1.49	1.00 ± 1.25	2.50 ± 2.07	2.61	0.067
Ears	0.20 ± 0.63	0.40 ± 0.70	0.80 ± 1.03	1.40 ± 1.84	1.49	0.235
Neck	0.00 ± 0.00	0.60 ± 1.58	0.90 ± 1.45	1.78 ± 1.56	2.31	0.095
Chin	0.30 ± 0.95	0.40 ± 1.26	0.70 ± 1.25	**2.22 ± 1.79** ^§^	3.66	**0.022**
Moustache	0.10 ± 0.32	1.10 ± 2.8	0.11 ± 0.33	**4.60 ± 1.26** ^§^	23.99	**<0.0001**
Beard	0.20 ± 0.42	0.00 ± 0.00	0.22 ± 0.67	**5.00 ± 0.00** ^§^	335.78	**<0.0001**
Body Hair	0.80 ± 1.48	1.60 ± 2.37	0.44 ± 1.33	**4.80 ± 0.63** ^§^	12.48	**<0.0001**
Shoulders	0.40 ± 0.70	0.10 ± 0.32	1.40 ± 1.51	**2.80 ± 1.87** ^§^	7.31	**0.001**
Arms	0.50 ± 0.71	0.90 ± 1.52	1.50 ± 1.64	**2.30 ± 1.83** ^§^	3.26	**0.033**
Hands	0.00 ± 0.00	0.90 ± 1.37	0.90 ± 1.37	**3.20 ± 1.93** ^§^	8.86	**<0.0001**
Chest	0.50 ± 0.85	0.20 ± 0.42	**2.90 ± 2.13** ^∞^	**2.80 ± 1.93** ^∞^	9.48	**<0.0001**
Breast	0.00 ± 0.00	1.00 ± 1.56	**4.90 ± 0.32** ^£^	**2.00 ± 2.40** ^∞∞^	18.24	**<0.0001**
Stomach	0.50 ± 1.27	0.10 ± 0.32	1.10 ± 0.32	1.30 ± 1.64	1.94	0.141
Belly	0.80 ± 1.23	1.20 ± 1.48	**2.70 ± 1.77** ^∞^	**2.40 ± 1.90** ^∞^	3.68	**0.021**
Genitals	0.20 ± 0.42	0.30 ± 1.08	**4.50 ± 0.85** ^∞^	**4.40 ± 1.35** ^∞^	49.76	**<0.0001**
Buttocks	0.40 ± 0.84	1.00 ± 1.34	**2.60 ± 1.65** ^∞^	**3.10 ± 1.73** ^∞^	7.69	**<0.0001**
Hips	0.10 ± 0.32	11.10 ± 1.29	**3.50 ± 1.84** ^££^	**2.40 ± 2.07** ^∞∞^	13.23	**<0.0001**
Thighs	**0.10 ± 0.32** ^¥^	2.40 ± 2.01	3.50 ± 1.58	2.40 ± 1.71	11.93	**<0.0001**
Knees	0.00 ± 0.00	1.50 ± 2.01	0.80 ± 0.92	**2.00 ± 2.05** ^$$^	4.07	**0.014**
Legs	0.30 ± 0.95	1.60 ± 1.96	1.90 ± 1.37	2.00 ± 1.76	2.54	0.073
Ankles	0.20 ± 0.63	1.20 ± 1.87	0.80 ± 1.35	**1.90 ± 1.73** ^§^	3.19	**0.036**
Feet	0.20 ± 0.63	0.60 ± 0.84	1.20 ± 1.40	**3.10 ± 1.73** ^§^	7.92	**<0.0001**
Smell	0.20 ± 0.63	0.30 ± 0.48	0.60 ± 0.43	**2.70 ± 0.67** ^§^	9.82	**<0.0001**
Body sounds	0.00 ± 0.000	0.30 ± 0.48	0.50 ± 0.97	**2.50 ± 2.07** ^§^	9.08	**<0.0001**
Sweating	0.70 ± 1.16	1.00 ± 1.86	1.20 ± 1.40	**3.10 ± 1.97** ^§^	4.48	**0.009**
Blushing	0.20 ± 0.63	0.60 ± 0.84	1.60 ± 2.01	0.80 ± 1.55	1.38	0.267

^§^ transwomen vs. other groups; ^¥^ cismen vs. other groups; ^∞^ transwomen and transmen vs. ciswomen and cismen; ^∞∞^ transwomen vs. cismen; ^£^ transmen vs. other groups; ^££^ transmen vs. ciswomen and cismen. ANCOVA = Analysis of covariance. Boldfaced numbers highlight statically significant differences between groups.
